# Chalcogenide glass nanospheres with tunable morphology by liquid-phase template approach

**DOI:** 10.1016/j.isci.2023.106111

**Published:** 2023-02-02

**Authors:** Yue He, Ruolan Zhao, Yu He, Xinyu Chen, Guangming Tao, Chong Hou

**Affiliations:** 1 School of Optical and Electronic Information, Huazhong University of Science and Technology, Wuhan 430074, China; 2 Sport and Health Initiative, Optical Valley Laboratory and Wuhan National Laboratory for Optoelectronics, Wuhan 430074, China; 3 State Key Laboratory of Material Processing and Die & Mould Technology, School of Materials Science and Engineering, Huazhong University of Science and Technology, Wuhan 430074, China; 4 Research Institute of Huazhong University of Science and Technology in Shenzhen, Shenzhen, 518063, China

**Keywords:** Mechanical processing, Thermal property, Materials design

## Abstract

Chalcogenide glass (ChG) with unique material properties has been widely used in mid-infrared. Traditional ChG microspheres/nanospheres preparation usually uses a high-temperature melting method, in which it is difficult to accurately control the size and the morphology of the nanospheres. Here, we produce nanoscale-uniform (200–500 nm), morphology-tunable, and arrangement-orderly ChG nanospheres from the inverse-opal photonic crystal (IOPC) template by the liquid-phase template (LPT) method. Moreover, we refer to the formation mechanism of nanosphere morphology as the evaporation-driven self-assembly of colloidal dispersion nanodroplets within the immobilized template and find that the concentration of ChG solution and the pore size of IOPC are the key to control the morphology of the nanospheres. The LPT method is also applied to the two-dimensional microstructure/nanostructure. This work provides an efficient and low-cost strategy for the preparation of multisize ChG nanospheres with tunable morphology and is expected to find various applications in mid-infrared, optoelectronic devices.

## Highlights

• A liquid-phase template method to prepare chalcogenide glass nanospheres was proposed
• The nanoscale-uniform chalcogenide glass nanospheres possessed tunable morphology
• The formation mechanism of nanosphere morphology was discussed

## Graphical abstract

**Figure:**
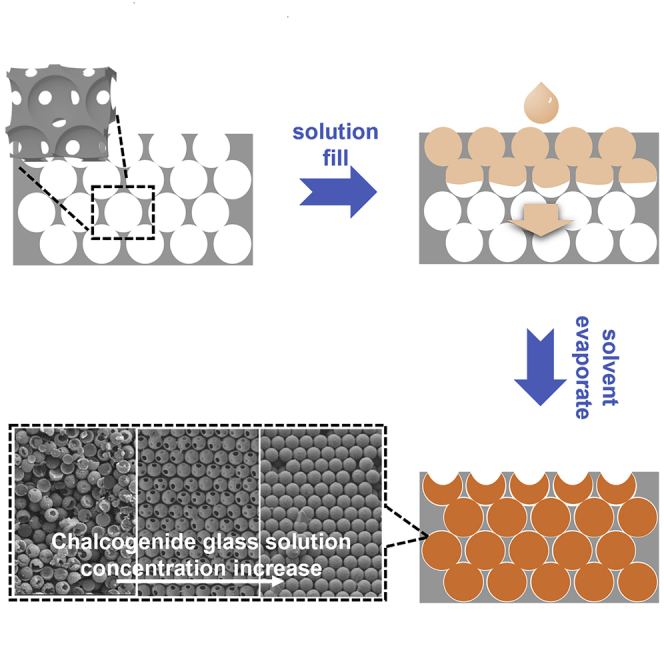

## Introduction

Common microspheres/nanospheres often exhibit unique properties and are widely used in areas such as optical devices, drug delivery, and biochemical catalysis, benefiting from their limited dimension and the flexible preparation methods, as well as their tailored morphologies and chemical compositions.^1^^,^^2^^,^^3^^,^^4^ Meanwhile, among the mid-infrared materials, chalcogenide glass (ChG) has become an ideal material for manufacturing new micro-nano photonic devices, due to their excellent mid-to-far infrared transmission performance, high refractive index, nonlinear refractive index, ultrafast nonlinear optical response, etc.^5^^,^^6^^,^^7^ ChG microspheres/nanospheres are usually prepared by melting method, in which process the ChG preforms (powder or fiber, etc.) turn into a molten state after reaching the glass transition temperature and a regular spherical shape is formed driven by the surface tension/Rayleigh instability.^8^^,^^9^^,^^10^ The melting method has certain limitations: 1) it is difficult to prepare the microspheres/nanospheres with complex architecture (e.g., porous microspheres/nanospheres) and 2) it often requires a high-temperature environment. The liquid-phase method is frequently used to realize complex architecture in microspheres/nanospheres, in which the particles are produced through nucleation, co-precipitation or sol-gel, etc.^11^ However, it still has problems such as low controllability over nanospheres size, complex preparation process, etc. Another useful method, called template method,^12^ could use polymer templates to prepare nanospheres of different materials (metals, polymers, and oxides)^13^ or use patterned template to produce nanostructure with different shapes.^14^ This method has advantages on the versatile preparation process, controllability over size and shape, and wide material compatibility.

In this article, we use a method, called liquid-phase template (LPT), which combines the merits of liquid-phase method and template method to produce uniform ChG nanospheres. In this LPT method, the ChG solution is filled into the IOPC template which has three-dimensional uniform pores, and after the solvent is completely evaporated, the ChG nanospheres with uniform size are obtained. The nanospheres with tunable morphology could be prepared using this method by tuning the ChG solution concentration and the template pore diameter. The LPT method is also versatile, as ChG “nano-bowls” and ChG micro-discs could be produced in different templates.

## Results and discussion

### Preparation of the ChG nanospheres

**Figure 1:**
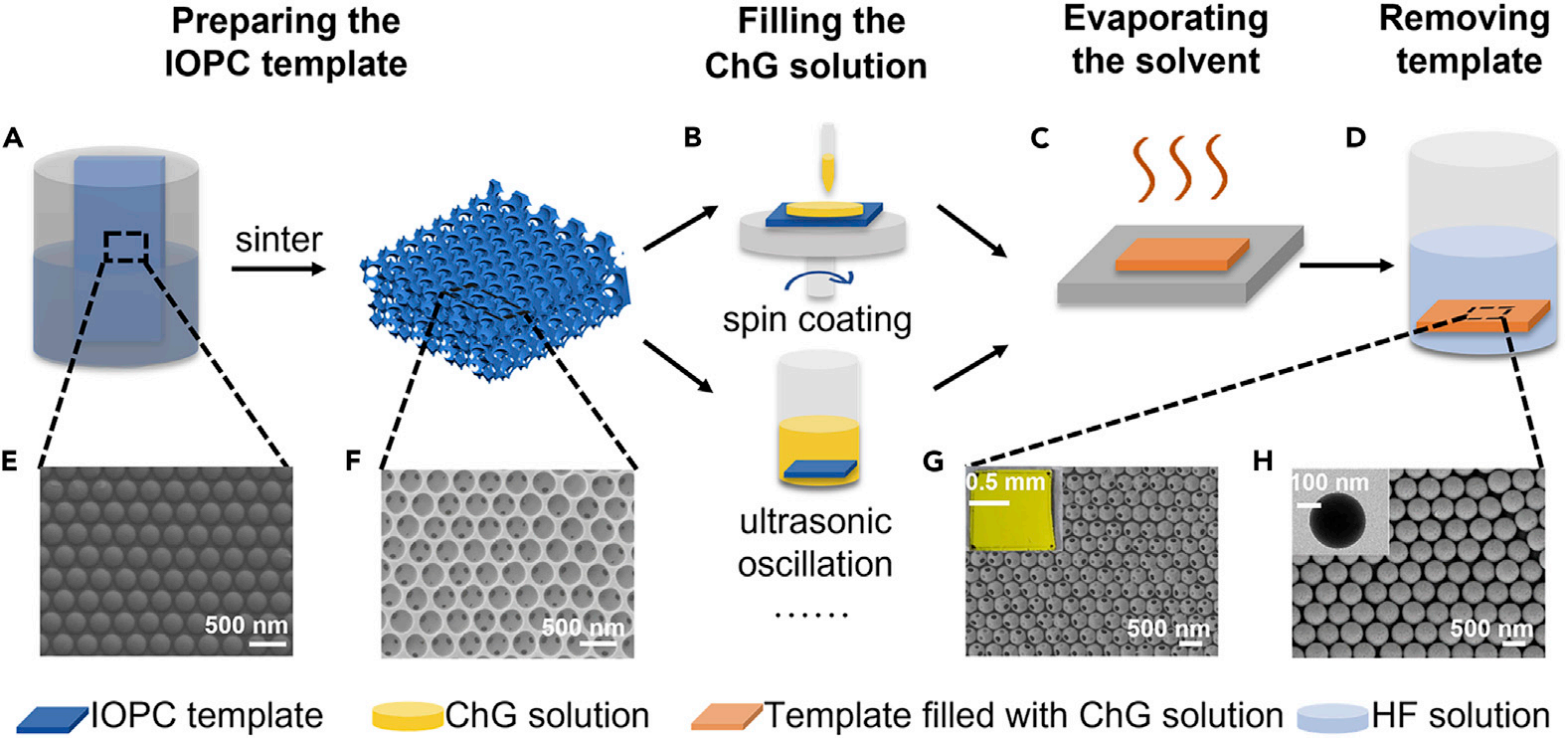
ChG nanospheres prepared by the LPT method (A–D) Schematic illustration of the fabrication process of ChG nanospheres: preparing the IOPC template, filling the ChG solution, evaporating the solvent, and removing template. (E and F) SEM images of the IOPC template morphology before and after removing the PS-NPs array, respectively. (G and H) SEM images show two kinds of ChG nanospheres OPC film. Figure 1G inset shows the optical picture of the film. Figure 1H inset shows the TEM image displaying the roundness of the nanospheres.

The process of LPT method includes preparing the IOPC template, filling the ChG solution, evaporating the solvent, and removing the template (Figures 1A–1D). Specifically, evaporation co-assembly is performed to prepare the opal photonic crystal (OPC) film,^15^ which consists of polystyrene nanoparticles (PS-NPs) opal film and silica gel matrix material distributed uniformly. The scanning electron microscopy (SEM) images of PS-NPs film show the highly ordered structure (Figure 1E). After high-temperature sintering, the IOPC SiO_2_ template is obtained (Figure 1F), with the uniform pore size inside determined by PS-NPs. Owing to the high adhesion to the substrate and exceptional mechanical performance which are further improved by the annealing process, this template is able to keep its integrity during the solution-filling process.^16^ Subsequently, the bulk ChG (As_30_S_70_) is dissolved in highly volatile amines (N-propylamine) forming nano-colloidal solutions^17^ and filled into the IOPC template by spin coating and ultrasonic oscillation method. Next, the IOPC template is heated for 1h at 80°C to evaporate the amines solvent and annealed at 120°C for 6 h in vacuum to make sure the amines solvent is totally evaporated. After the annealing process, the ChG nanospheres OPC is formed inside the IOPC template (Figure S1). Finally, the IOPC template is etched off in hydrofluoric acid, and a large area of uniform ChG nanospheres OPC film is obtained, which shows different morphologies (Figures 1G and 1H). Owing to the connection among the ChG nanospheres (Figure S2) and the ChG being generally inert to acids, the ChG nanospheres remain on the substrate and keep the OPC structure.^18^^,^^19^

### Characterization of the ChG nanospheres

**Figure 2:**
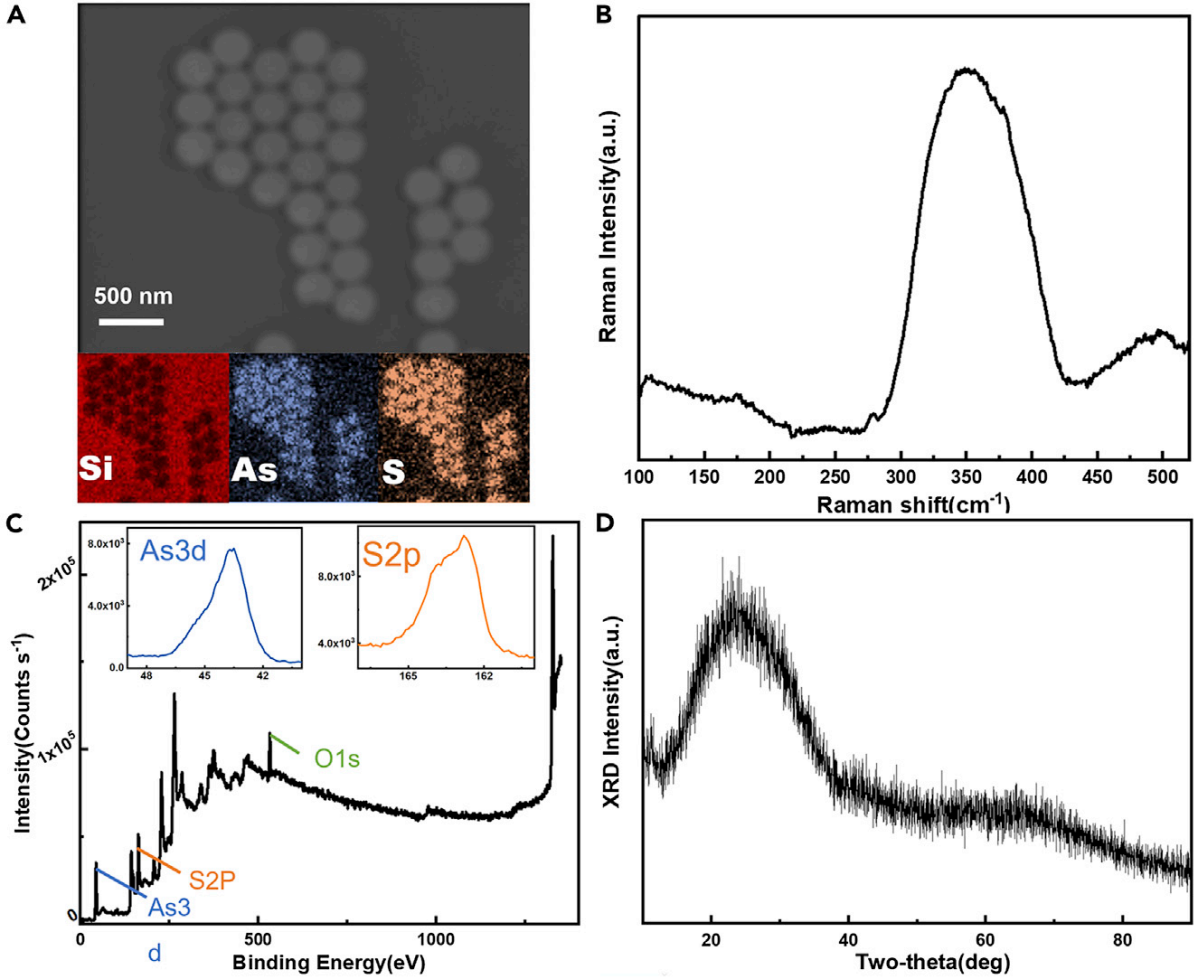
Characteristics of the ChG nanospheres (A–D) show EDS, Raman spectrum, XPS, and XRD characterization of ChG nanospheres, respectively.

The energy-dispersive spectrometer (EDS) analysis shows that the elemental composition of nanospheres prepared by the LPT method is As and S, which is consistent with that of bulk glass (Figure 2A). The Raman spectrum is collected after the ChG nanosphere OPC film gets irradiated by the 473 nm laser (Figure 2B). It has a wide Raman vibration band at 350 cm^−1^, which belongs to the As-S bond and further confirms the nanosphere composition. In addition, the spectrum has a peak at 495 cm^−1^ which hints that nanospheres have a composition close to As_36_S_64_, instead of As_30_S_70_ in the bulk glass.^20^ The loss of sulfur could happen during the dissolving process, in which some sulfur precipitates out of the solution due to the dissolving limit.^21^ The X-ray photoelectron spectroscopy (XPS) characterization results (Figure 2C) also confirm that the composition of nanospheres is As and S and show an unnecessary O1s peak. The reason for this may be that the sulfur gets oxidized in the heating and annealing process and brings in the O element. The X-ray diffraction (XRD) confirms that the nanospheres are amorphous after being annealed at 120°C for 6 h (Figure 2D). We also characterize the reflection spectra of the ChG OPC films (Figure S3). The reflection peak exhibits blue-shift trend with increasing incident angle, which is consistent with the properties of photonic crystals. More importantly, the reflection peak could be tuned by changing the size of the nanospheres and ChG solution mass fraction. This photonic property makes them suited to light relevant applications, such as sensing, lasers, energy storage, and so forth. For example, simply altering the refractive index contrast by changing one of the materials with a material of different refractive index will produce a shift in the stop band of the photonic structure, which is ideal for sensor applications to monitor the temperature, detect the gas, etc.^22^^,^^23^; by embedding an optical gain medium (dye molecules or quantum dots) in the three-dimensional structures, a high refraction index contrast between ChG nanospheres and air can realize low-threshold lasing at the band edge of photonic crystals, and the lasing wavelength can be controlled by simply changing the size of the nanospheres.^24^

**Figure 3:**
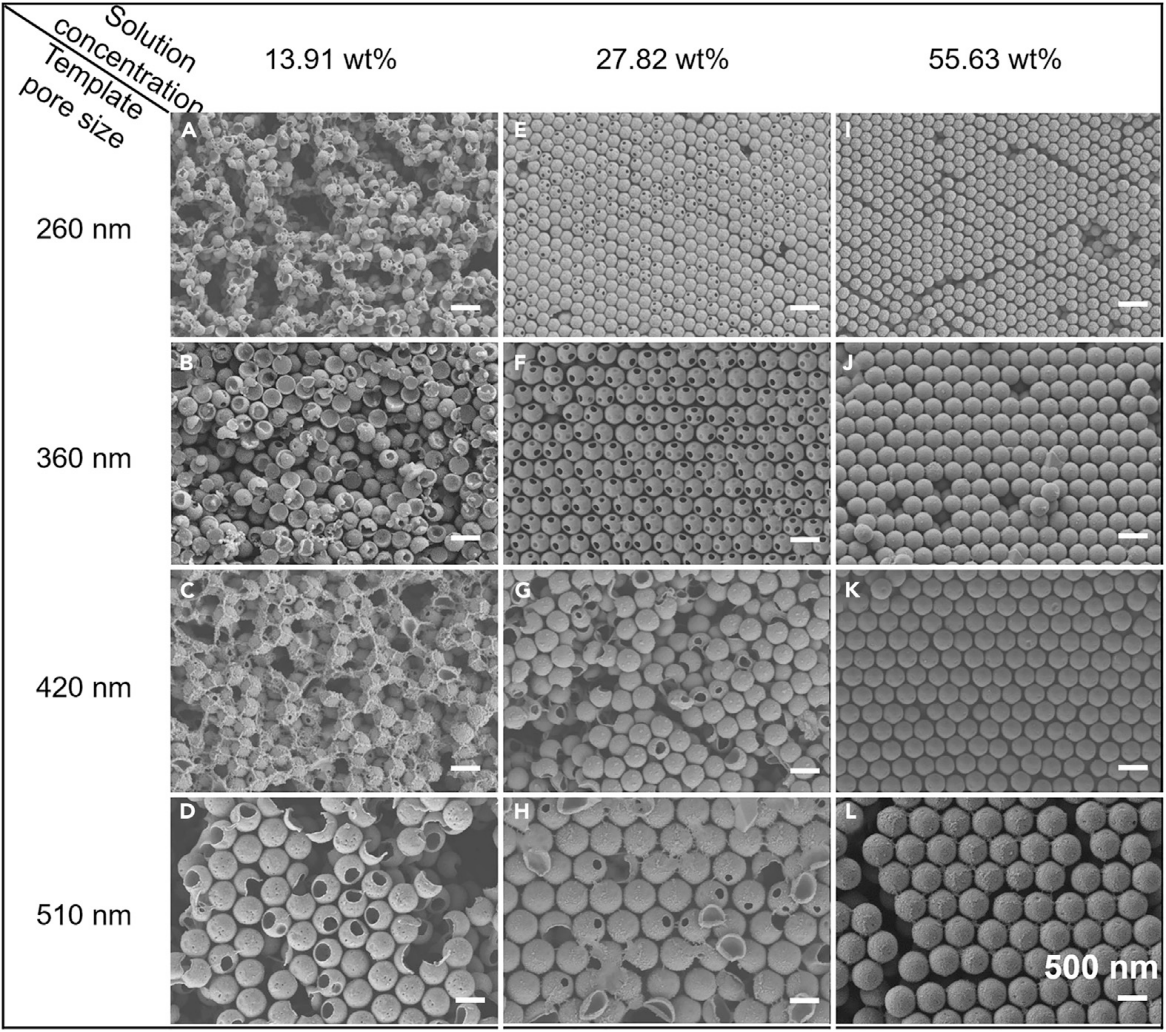
Nanospheres morphology diagram determined by ChG solution concentration and the pore size of IOPC template (A–D) The SEM images show morphology of the ChG nanospheres prepared by low ChG solution mass fraction (13.91 wt %) and the template pore size ranging from 260 to 510 nm. (E–H) The SEM images show morphology of the ChG nanospheres prepared by higher ChG solution mass fraction (27.82 wt %) and the template pore size ranging from 260 to 510 nm. (I–L) The SEM images show morphology of the ChG nanospheres prepared by the highest ChG solution mass fraction (55.63 wt %) and the template pore size ranging from 260 to 510 nm. All the scale bar is 500 nm.

An important feature of the LPT method is that ChG nanospheres with different morphologies could be flexibly prepared by adjusting the concentration of the ChG precursor solution and the pore size of the IOPC template. Figure 3 shows the nanospheres morphology diagram under different IOPC template pore sizes and ChG mass fractions. At a low ChG solution mass fraction (13.91 wt %), the nanospheres are mostly semisphere or hemishell under any of the template pore sizes, and the OPC structure of the ChG nanospheres film is mostly broken (Figures 3A–3D). As the mass fraction increases to 27.82 wt %, “large holes” appear on the surface of the ChG nanospheres, which correspond to the pore interconnectivity points in the IOPC template (Figures 3E–3H). When the mass fraction is further increased to 55.63 wt %, the “large holes” disappear and the complete ChG nanospheres are fabricated (Figures 3I–3L). The cross-section image of the nanospheres OPC film shows a consistent result (Figure S4). In the meanwhile, comparing sphere size over pore size, it is not difficult to find that the size of the ChG nanospheres gradually increases along with the pores. It is worth noting that when produced from the same template, the nanospheres prepared at high mass fraction are smaller than those prepared at low mass fraction, which hints that the inside structure of nanospheres prepared at low mass fraction is loose and porous (Figure S6). This unique structure makes the nanospheres possess large surface area, low density, and high loading capacity. These properties enable nanospheres to have a large variety of applications, including microcontainers/nanocontainers, environmental remediation, biomedicine, and more. For example, void space existing inside or on the porous spheres can provide the "microenvironment" for many chemicals such as dye molecules, organic drugs, and inorganic nanoparticles, enabling controlled molecule release or drug delivery;^25^^,^^26^ owing to their large surface area, porous nanospheres have strong affinity toward dyes, organic pollutants, heavy metal ions, etc., making them ideal for applications of water purification and environmental remediation.^27^

### Discussion of ChG nanospheres-forming mechanism

**Figure 4:**
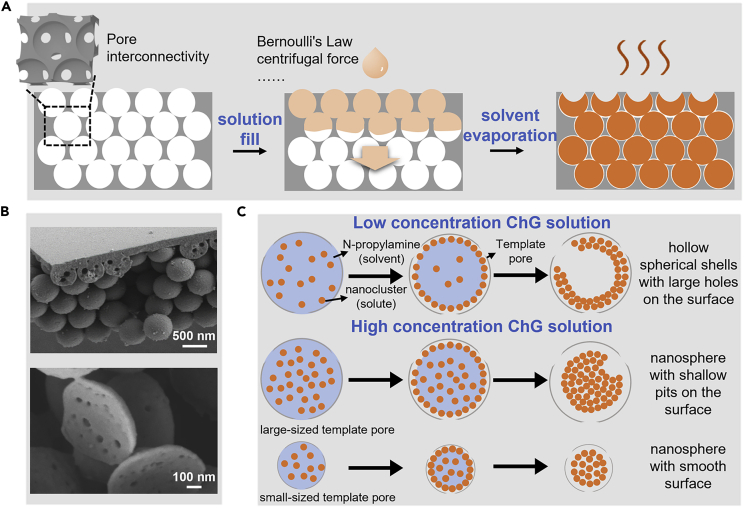
Schematic of ChG nanospheres preparation principle (A) Pattern-formation process is simply divided into two steps, including solution fill process and solvent evaporation process. (B) SEM image of the porous structure inside ChG nanospheres. (C) The formation mechanism of ChG nanospheres with tunable morphology.

Based on the phenomenon that different solution concentrations and pore sizes of the template could prepare tunable morphology, the ChG nanospheres-forming mechanism is discussed. Figure 4A shows the two critical steps of making ChG nanospheres: 1) ChG solution filling into the template and 2) ChG solvent being evaporated to get the ChG nanospheres. During the ChG solution filling process, the IOPC template ensures the filling rate of ChG solution with up to 12 interconnected points between neighboring pores. To further increase the filling rate, different methods (spin coating, ultrasonic oscillation, etc.) are used to get a better filling quality (Figure S5).

During the solvent evaporation process, the ChG solution is gradually broken into small nanodroplets,^28^ whose size are limited by the pores of the template. The subsequent evaporation process of the nanodroplets can be referred to as the evaporation-driven self-assembly of colloidal dispersion nanodroplets within the immobilized template. Figure 4C illustrates the process of ChG nanodroplets turning into ChG nanospheres: as the solvent is evaporated, the nanoclusters (average 1–4.5 nm) in the nanodroplets aggregate to form nanospheres with porous structure and tunable morphology.^21^^,^^29^^,^^30^ Figure 4B demonstrates the porous structure inside the ChG nanospheres due to the gas generated by the chemical reaction during the evaporation process.^31^

The concentration of the ChG solution and the pore size of the template play an important role in tuning the ChG nanosphere morphology, and Figure 4C illustrates the evaporation-driven self-assembly of colloidal dispersion nanodroplets within the immobilized template in three cases and fully agrees with the experimental phenomenon in Figure 3. The nanodroplets initially behave like pure liquids and shrink isotropically during evaporation but eventually form a viscoelastic shell of dense nanoclusters on their surface.^32^ This is due to a thermophoretic force that originates from the temperature gradient at the droplet surface and thus also causes the concentration gradient of the solute in the droplet.^33^ When using low-concentration ChG solutions, the nanoclusters on the nanodroplet surface are dominated by the concentration gradient force pointing to the sphere center due to the solute concentration gradient. In addition, they are also subjected to the outward electrostatic repulsion force provided by the adjacent surface clusters and the inner nanoclusters of the nanodroplets. For the nanocluster located at the pore junction of the template, the electrostatic repulsion force is not enough to balance with the concentration gradient force, resulting in collapse. The nanoclusters not at the pore junction of the template are usually subjected to the outward adhesion force from the template, which is more stable than the nanoclusters at the pore junction of the template. Therefore, the nanodroplets formed hollow spherical shells with large holes at the pore junction of the template (Figures 3A–3H). In the case of high-concentration ChG solution, the concentration gradient force toward the sphere center of the surface nanoclusters due to the concentration gradient of the solute will be relatively reduced, and the electrostatic force provided by the nanoclusters inside the nanodroplet will be larger because of the inherent constraints of availability of space.^32^ In the large-sized template pore, the nanoclusters located at the pore junction of the template also occasionally collapsed at the early stage of solvent evaporation, resulting in shallow pits on the surface of the prepared nanospheres (Figure 3L). While in the small-sized template pore, the concentration gradient force toward the sphere center of the surface nanoclusters further reduced, the decrease of the nanodroplet radius increases the outward electrostatic repulsion provided by the adjacent surface and the inner nanoclusters of the nanodroplet. Therefore, the surface nanoclusters maintain the quasi-steady state during the whole solvent evaporation process, and the smooth surface nanospheres are finally prepared (Figure 3I). The evaporation-driven self-assembly of colloidal dispersion nanodroplets is relatively more stable and slower when using a high-concentration ChG solution. Therefore, the diameter of the nanospheres prepared with a high-concentration of ChG solution is slightly smaller than the spherical shells prepared with a low concentration of ChG solution (Figure S6). The detailed analysis of the evaporation-driven self-assembly of colloidal dispersion nanodroplets within the immobilized template is in method details.

### Process versatility

**Figure 5:**
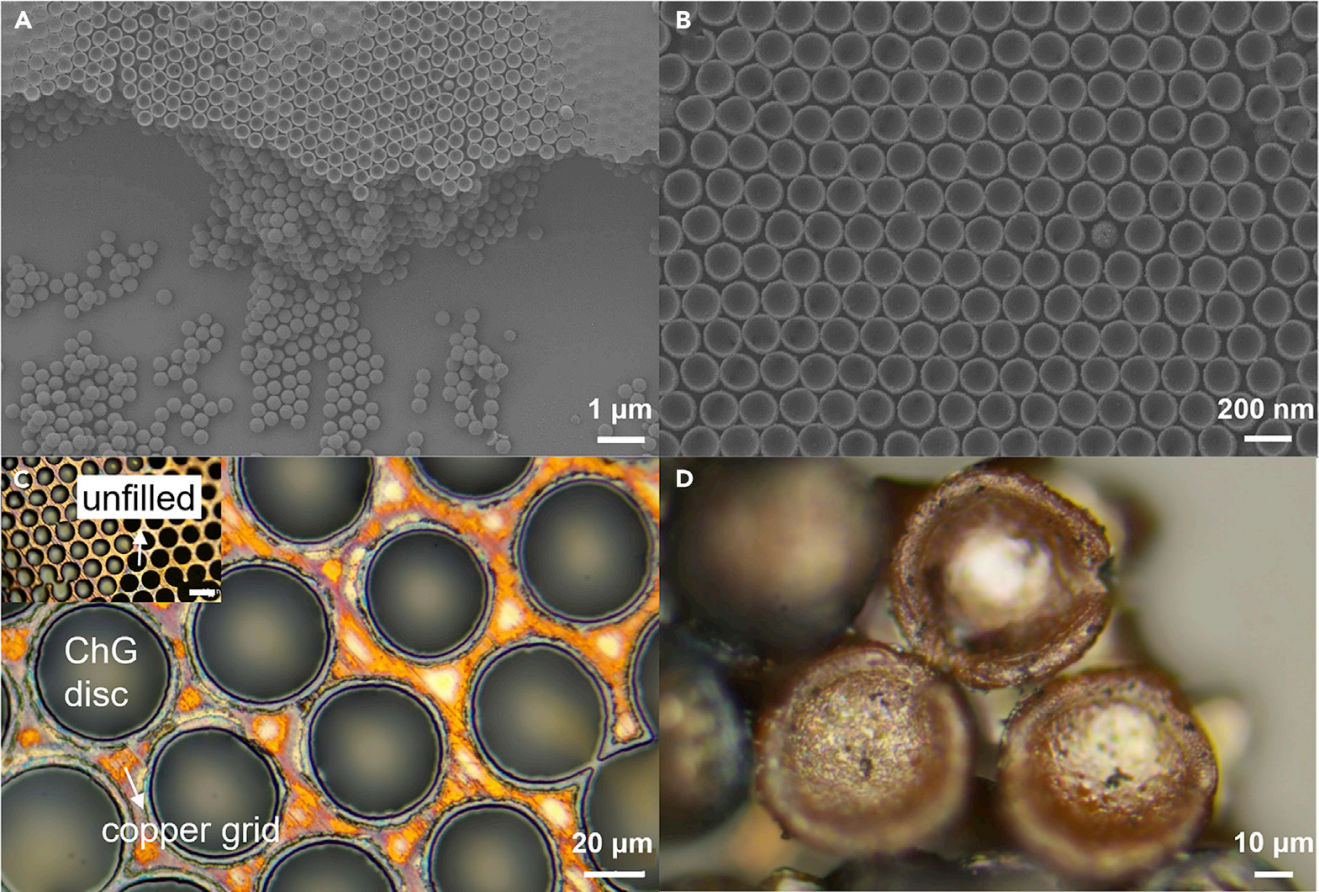
Process versatility (A) SEM image shows a “bowl-like” nanostructure at the uppermost layer of the ChG nanospheres OPC film. (B) shows a high-magnification image. (C) Optical image shows the ChG disc prepared by copper mesh template. The illustration shows a low-magnification image. The scale bar in the illustration is 50 μm. (D) shows a high-magnification image.

The LPT method could be extended to prepare ChG with other microstructures/nanostructures. For example, on the top of the ChG nanospheres OPC film, the "bowl-like" nanostructure is formed because the uppermost layer of the IOPC template is a semi-enclosed structure; the “bowl-like” ChG nanostructure has a diameter of around 150 nm (Figures 5A and 5B). The LPT method is also used with a copper mesh to produce ChG micro-discs. The copper mesh, as the template in this method, is suspended, and the ChG solution is dripped on top of it (Figure S7). After the solvent is completely evaporated, ChG discs with diameter of around 60 μm are obtained by etching off the copper mesh with hydrochloric acid (Figures 5C and 5D).

In summary, we demonstrate an LPT method to realize ChG nanospheres with tunable size and morphology. We discuss the ChG nanosphere-forming mechanism and propose a model to explain the morphology of the nanospheres. This method is suitable for not only three-dimensional templates (such as IOPC) but also two-dimensional templates (such as copper mesh). This work provides an efficient and low-cost strategy for the preparation of multisize ChG nanospheres with tunable morphology and is expected to find a variety of applications in mid-infrared, optoelectronic devices.

### Limitations of the study

ChG microspheres of different components have not been prepared, and the universality of the LPT method has not been verified. In addition, when preparing ChG nanospheres from low concentration of ChG solutions, the surfaces are often covered with a thin ChG film, which has a certain impact on the expression of photonic crystal properties.

## STAR★Methods

### Key resources table

**Table:** 

REAGENT or RESOURCE	SOURCE	IDENTIFIER
**Chemicals, peptides, and recombinant proteins**		

Chalcogenide glass (ChG)	Hangzhou Changbo infrared technology Co., Ltd., China	N/A
Ethanol	Aladdin	CAS number: 64-17-5
Tetraethyl orthosilicate (TEOS)	Aladdin	CAS number: 78-10-4
Ammonium persulfate (APS)	Aladdin	CAS number: 7727-54-0
Styrene (St)	Aladdin	CAS number: 100-42-5
Propylamine	Aladdin	CAS number: 107-10-8
Hydrochloric acid (HCl)	Sinopharm Group Chemical Reagent Co. Ltd., China	CAS number: 7647-01-0
Hydrofluoric acid (HF)	Aladdin	CAS number: 7664-39-3

**Software and algorithms**		

Origin 2019	OriginLab	www.originlab.com
Nano Measurer	Department of Chemitry, Fudan University	https://nano-measurer.software.informer.com/

**Other**		

Scanning electron microscopy	ZEISS	Gemini
Raman spectra	HORIBA	Symphony Ⅱ
X-ray photoelectron spectroscopy	Thermo Fisher Scientific	ESCALAB 250Xi
XRD analyzer	Bruker	D8 ADVANCE
Metallographic microscope	Sunny Optical Technology (Group) Co., Ltd	CX40M
Spectrophotometer	Shimadzu	SolideSpec-3700

### Resource availability

#### Lead contact

Further information and requests for resources and reagents should be directed to and will be fulfilled by the lead contact, Chong Hou at chong@hust.edu.cn.

#### Materials availability

This study did not generate new unique reagents.

### Experimental model and subject details

This study does not use experimental methods typical in the life sciences.

### Method details

#### PS-NPs preparation

The PS-NPs was were synthesized by surfactant-free emulsion polymerization. 1.98–7.96 mL St, 0.144 g APS, 120 mL DI water were added into the flask, heated at 70°C for 20 h. Then the PS-NPs were cleaned by centrifugation and formulated as a PS-NPs suspension with the concentration of 0.125 wt %.

#### IOPC template preparation

The IOPC template was prepared by evaporation co-assembly. The mass ratio of standard TEOS solution was 0.1 M HCl: TEOS: Ethanol = 2:2:3. The silicon substrate was hydrophilically treated and vertically inserted into the co-assembled suspension which contained 20 mL PS-NPs suspension and 0.15 mL standard TEOS solution. The co-assembled suspension was evaporated at 65°C for 2–3 d to deposit a thin film on to silicon substrate. Then the film was sintered at 500°C for 5 h to form the IOPC template.

#### ChG nanospheres preparation

4 g ChG was added into 10/20/40 mL propylamine, and stirred until dissolved. 200 μL ChG solution was filled into IOPC template by spin coating once at 5000 rpm, 60 s (with low viscosity ChG solution) or spin coating twice at 4000 rpm, 60 s (with high viscosity ChG solution). Then it was heated at 80°C for 1 h and annealed at 120°C in vacuum for 6 h, and then it was immersed into HF for 30 min to remove the IOPC template.

#### Characterization

The SEM images and EDS images were obtained by ZEISS Gemini. The Raman spectrum was obtained by HORIBA Symphony Ⅱ. The XPS image was obtained by Thermo Fisher Scientific escalab 250XI (Power 150 W, operating voltage 14.8 KV, current 1.6 A). The XRD image was obtained by Bruker D8 ADVANCE. The Optical images using a metallographic microscope (CX40M, Sunny Optical Technology (Group) Co., Ltd). The spectrum of ChG OPC film at different angles was obtained by spectrophotometer (SolideSpec-3700, Shimadzu). The measured spectral range was 400–2500 nm, and the measured angle (between the incident light and the normal) range was 5°–65°, which were recorded every 10°.

#### The analysis of the evaporation driven self-assembly of colloidal dispersion nanodroplets within the immobilized template

The three chemical equations below show the detailed reaction process of ChG solution during dissolution, evaporation, and annealing:

(Equation 1) $$\left(3+x\right)As_{2}S_{3}+12xRNH_{2}\to 3As_{2}S_{3+x}{\left(RNH_{3}^{+}\right)}_{2x}+2xAs{\left(RNH\right)}_{3}↓$$

(Equation 2) $$As_{2}S_{3+x}{\left(RNH_{3}^{+}\right)}_{2x}\overset{80-90{}^{\circ}C}{\to }As_{2}S_{3+x}H_{2x}+2xRNH_{2}↑$$

(Equation 3) $$As_{2}S_{3+x}H_{2x}\overset{>130{}^{\circ}C}{\to }As_{2}S_{3}+xH_{2}S↑$$

The solvent evaporation process of the nanodroplets can be referred to as the evaporation driven self-assembly of colloidal dispersion nanodroplets within the immobilized template. In the first stage of evaporation, the nanodroplets initially behave like pure liquids and shrink isotropically during evaporation, but eventually form a viscoelastic shell of dense nano-clusters on its surface. This is consistent for nanodroplets of different concentrations and sizes, and the tunable morphologies of the final prepared ChG nanospheres mainly depend on the second stage of evaporation. As the model established in Figures S8 and S9, the nanoclusters in the nanodroplet surface are first subjected to the electrostatic force ($F_{e}$) between the nanoclusters, which can be expressed by Coulomb’s law as in Equation 4:

(Equation 4) $$F_{e}=kQ_{1}Q_{2}/s^{2}$$

Where k is Coulomb’s constant, Q is the amount of charge, and s is the distance. Secondly, the surface nanoclusters are also subjected to the concentration gradient force ($F_{c}$) directed towards the nanodroplet center driven by the concentration gradient, which can be expressed by Equation 5:

(Equation 5) $$F_{c}=K\frac{d\rho }{dr}A$$

Where K is the concentration gradient force coefficient, ρ is the local density of the nanocluster, r is the distance from the center of the nanodroplet, $\frac{d\rho }{dr}$ is the particle concentration gradient, and A is the projection of the concentration potential region perpendicular to the direction of the force. In addition, the surface nanoclusters are subject to a relatively small adhesion force ($F_{a}$) provided by the template, which is proportional to the viscosity of the nanodroplet (V). As shown in Figure S10, the viscosity of ChG solution is proportional to its concentration (C), so the adhesion force is proportional to the concentration of ChG solution, that is, $F_{a}\propto V\propto C$.

When using low concentration ChG solutions, the concentration gradient force ($F_{c}$) on the surface nanoclusters dominates due to the large concentration gradient. For the nanoclusters located at the pore junction of the template, the outward electrostatic repulsion force $\left(F_{e\left(1\right)}\;\cos\;\theta +F_{e\left(2\right)}\;\cos\;\theta +F_{e\left(3\right)}\right)$ is insufficient to balance with the larger concentration gradient force ($F_{c}$), resulting in collapse. The nanoclusters not at the pore junction of the template are usually subject to the outward adhesion force from the template, which is more stable than the nanoclusters at the pore junction of the template. Therefore, the nanodroplets formed hollow spherical shells with large holes at the pore junction of the template.

In the case of high-concentration ChG solution, the concentration gradient force ($F_{c}$) will be relatively reduced due to the smaller concentration gradient. Conversely, $F_{e\left(3\right)}$ provided by the nanoclusters inside the nanodroplet will be larger because of the inherent constraints of availability of space. In the large-sized template pore, the nanoclusters located at the pore junction of the template also occasionally collapsed slightly, resulting in shallow pits on the surface of the prepared nanospheres. While in the small-sized template pore, the concentration gradient force ($F_{c}$) further reduced. In addition, $\theta^{\prime }$ will be smaller than θ due to the reduced diameter of the nanodroplets, resulting in the larger outward electrostatic repulsion force $\left(F_{e\left(1\right)}\;\cos\;\theta^{\prime }+F_{e\left(2\right)}\;\cos\;\theta^{\prime }+F_{e\left(3\right)}\right)$. Therefore, the surface nanoclusters maintain the quasi-steady state during the whole solvent evaporation process, and the smooth surface nanospheres are finally prepared.

### Quantification and statistical analysis

Data were expressed as mean ± SEM (standard error of mean).

## Data Availability

• All data reported in this paper will be shared by the lead contact upon request. • This article does not report original code. • Any additional information required to reanalyze the data reported in this article is available from the lead contact on request.
