# Supplementary material for: Chalcogenide glass nanospheres with tunable morphology by liquid-phase template approach
Source: iScience. 2023 Feb 2;26(3):106111. doi: 10.1016/j.isci.2023.106111 (PMC9984555; doi:10.1016/j.isci.2023.106111)
Supplement: Document S1. Figures S1–S10 [file mmc1.pdf]

iScience, Volume *26*

Supplemental information

Chalcogenide glass nanospheres with tunable morphology by liquid-phase template approach

Yue He, Ruolan Zhao, Yu He, Xinyu Chen, Guangming Tao, and Chong Hou

Supplemental Figures

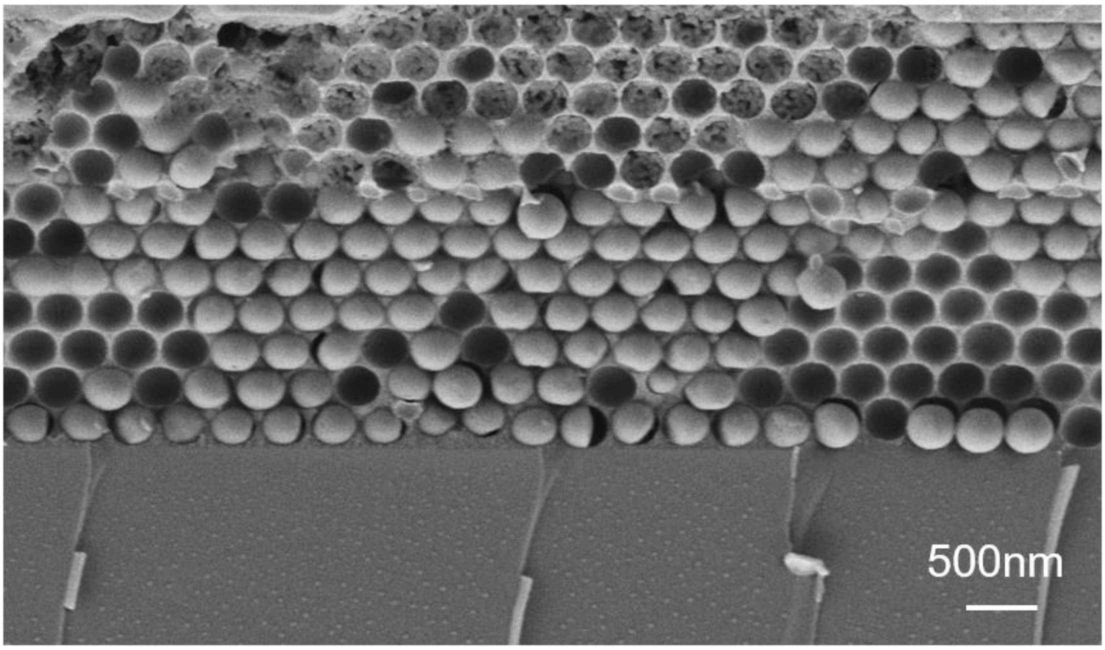

Figure S1. Cross-sectional SEM image of ChG nanospheres and IOPC SiO_2 template. Related to Figures 1e and 1f.

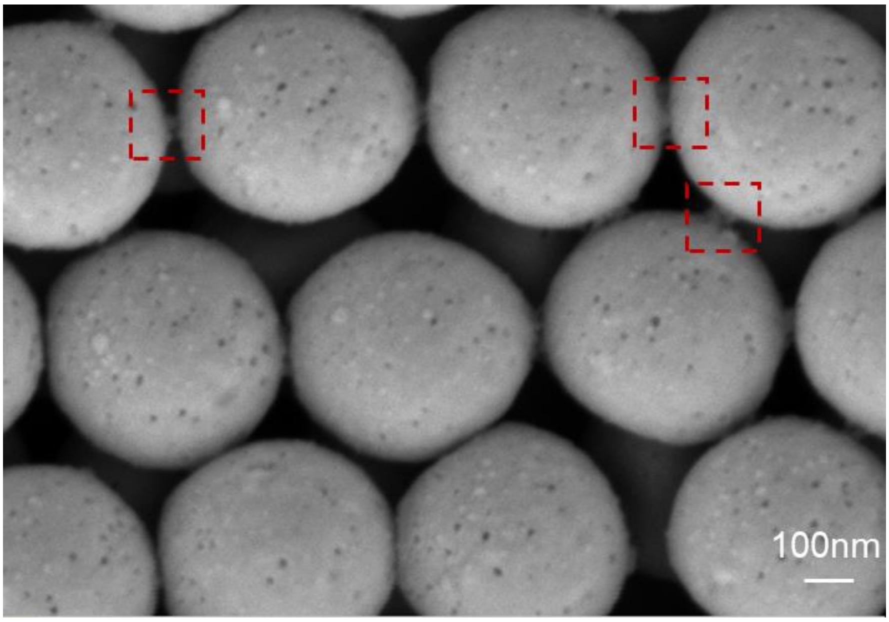

Figure S2. SEM image of ChG nanospheres OPC film. Related to Figure 1h.

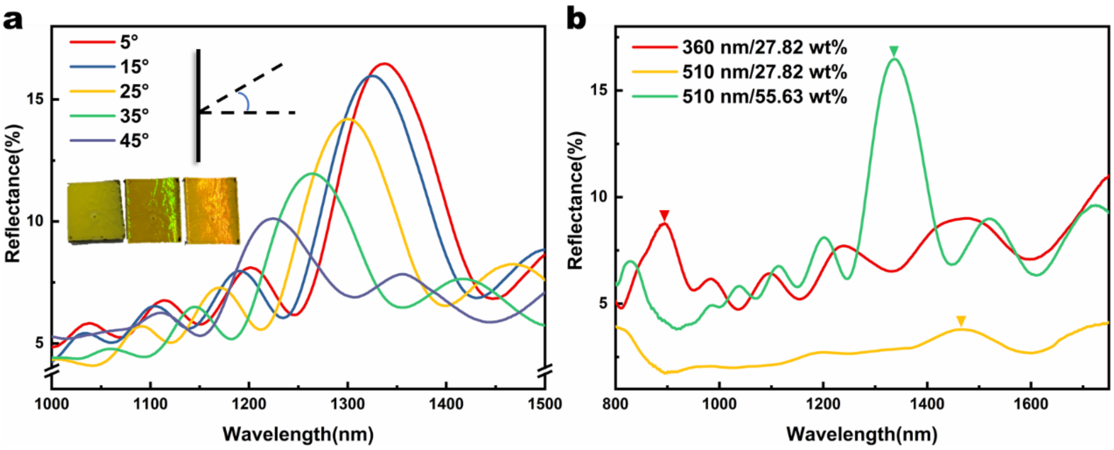

Figure S3. Reflectance characterization of ChG nanospheres OPC film. Related to Figure 2. (a) At different angles (510 nm/55.63 wt%). The illustrations are the film optical images from different angles. (b) At different template pore sizes and different ChG solution concentrations.

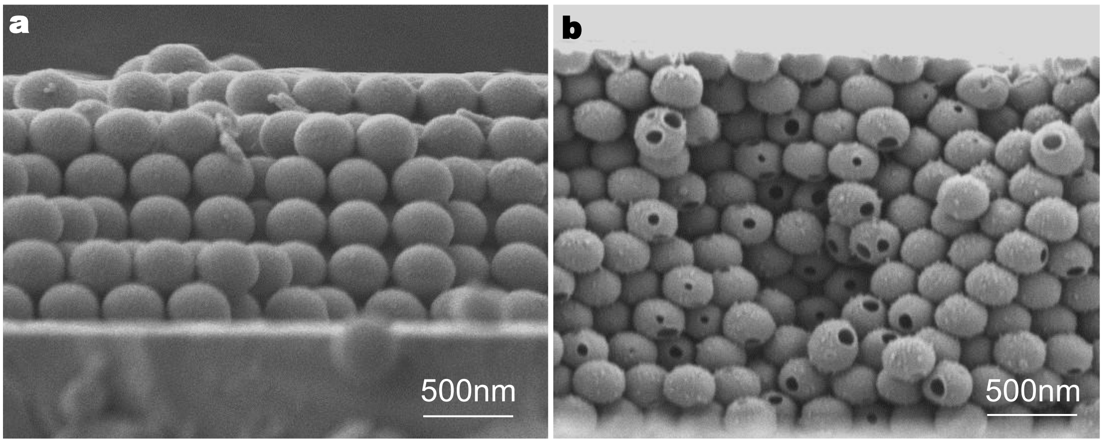

Figure S4. Cross-sectional SEM images of ChG nanospheres OPC film. Related to Figure 3. (a) Nanospheres prepared by high-concentration ChG solution. (b) Nanospheres prepared by low-concentration ChG solution.

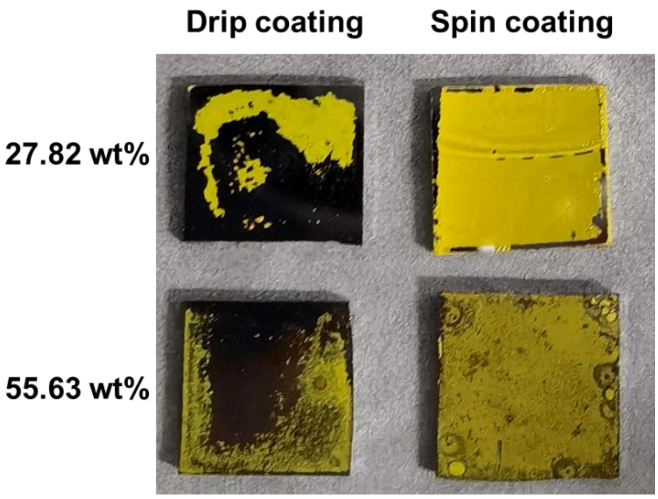

Figure S5. Optical image of ChG nanospheres OPC film. The filling rate of the drip-coating is compared with that of spin-coating. Each sample is infiltrated with 200 ul ChG solution in 60 s. Related to Figure 4a.

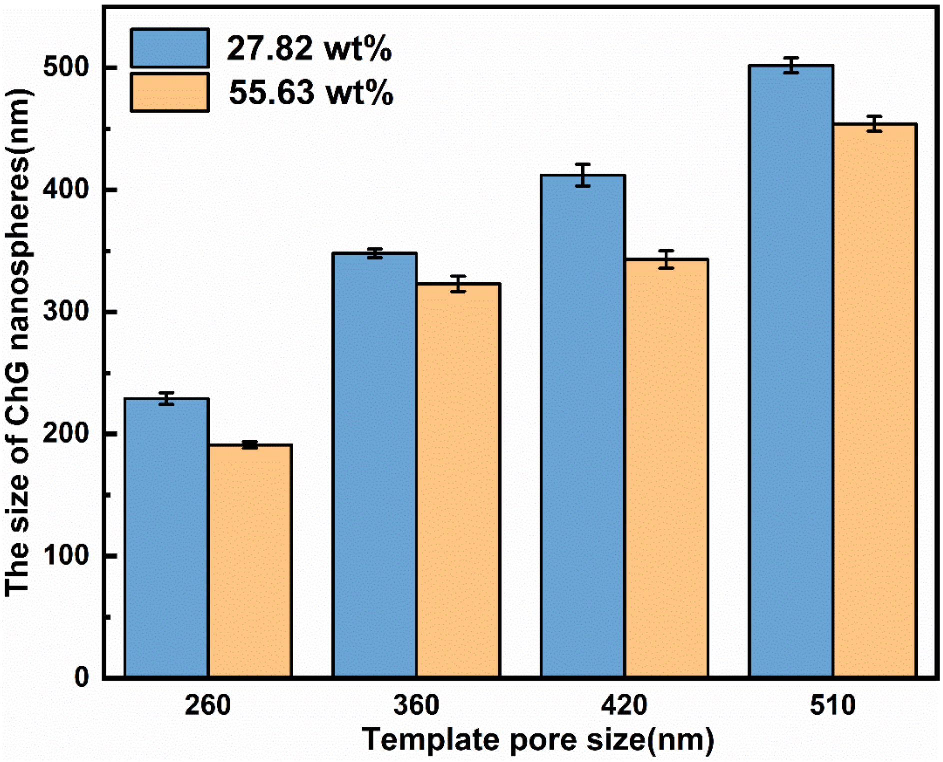

Figure S6. Size of GhG nanospheres prepared by the same mass fraction solutions from different template apertures. Data were expressed as mean ± SEM. Related to Figure 4c.

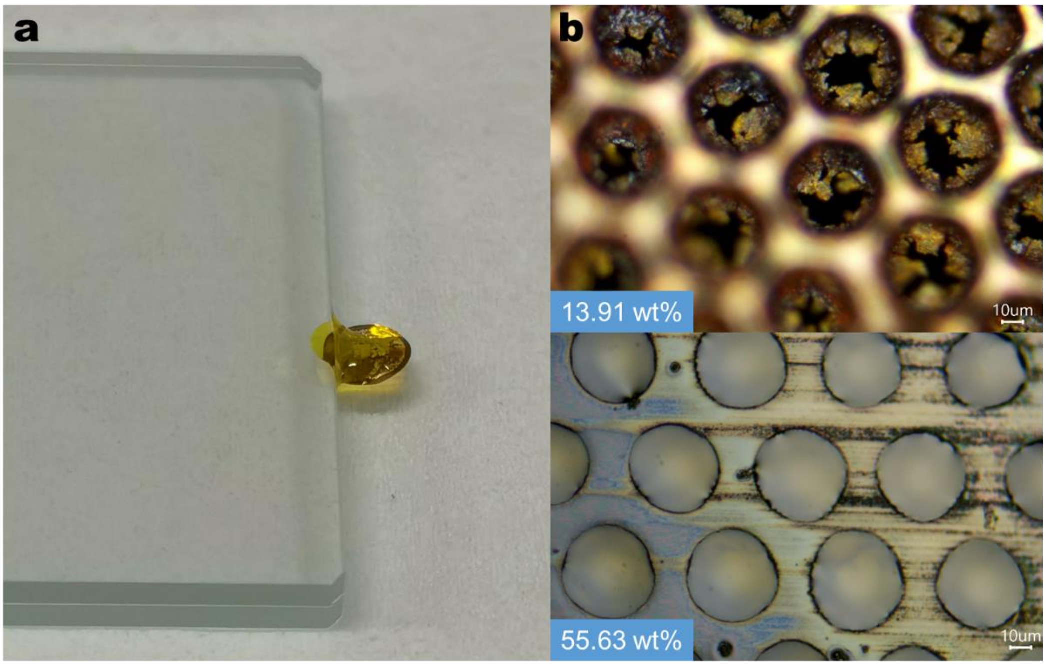

Figure S7. Preparation of ChG disc. Related to Figure 5. (a) Digital photograph of a simple device for preparing the ChG disc. (b) Optical images of ChG disc prepared by different ChG solution concentrations. Similar to the ChG nanospheres, ChG discs prepared at low-concentration were incomplete, and only a high-concentration solution could prepare relatively complete discs.

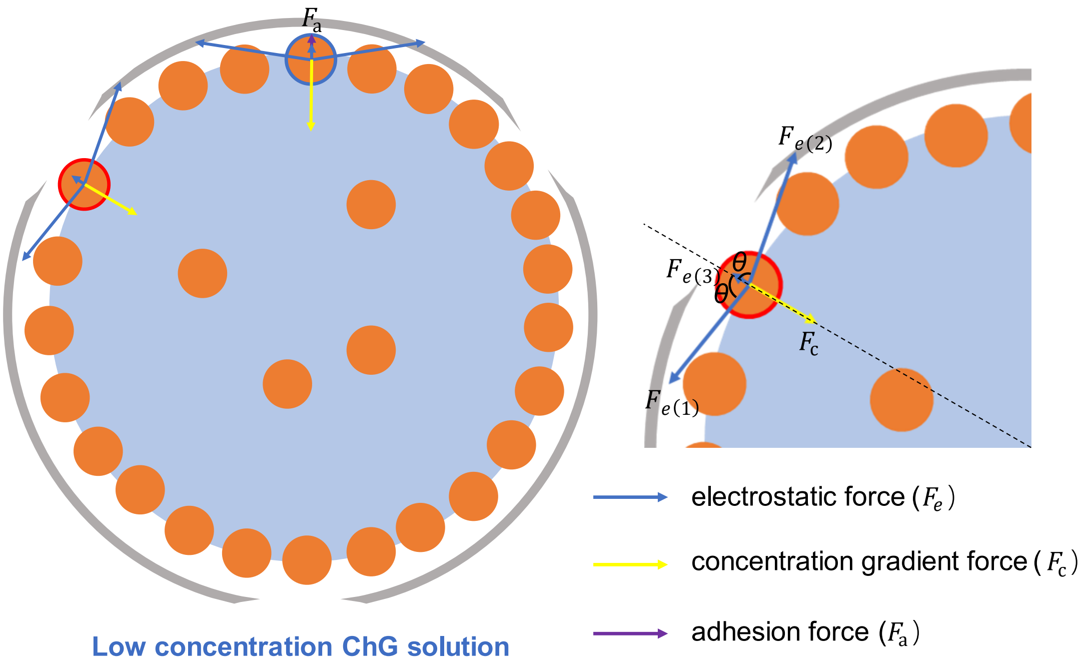

Figure S8. Mechanical analysis of surface nanoclusters using low-concentration ChG solution. Related to Figure 4c.

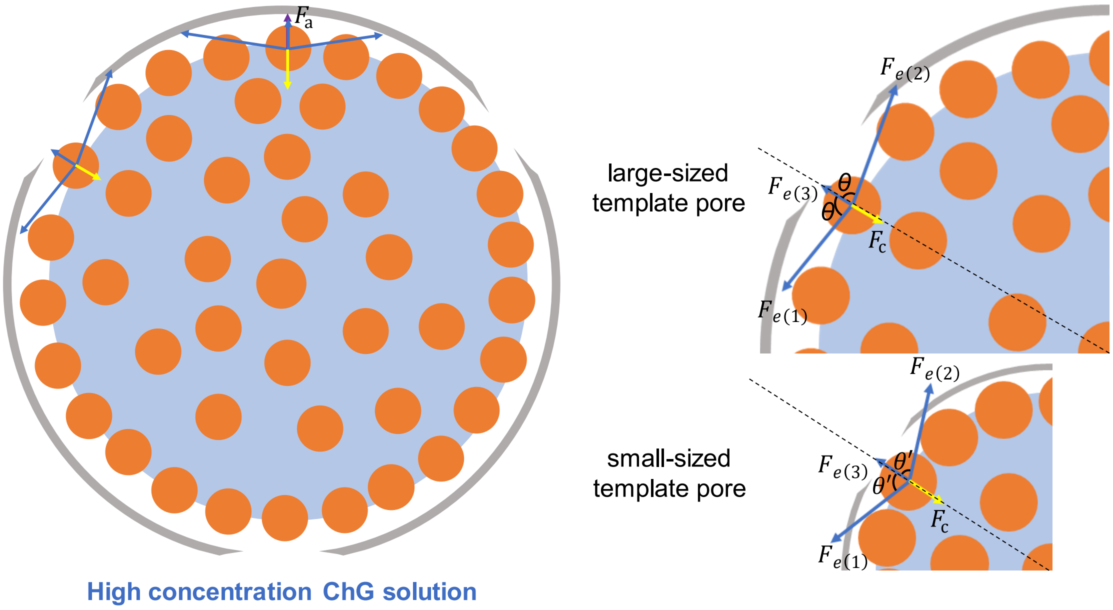

Figure S9. Mechanical analysis of surface nanoclusters in large-sized or small-sized template pore using high-concentration ChG solution. Related to Figure 4c.

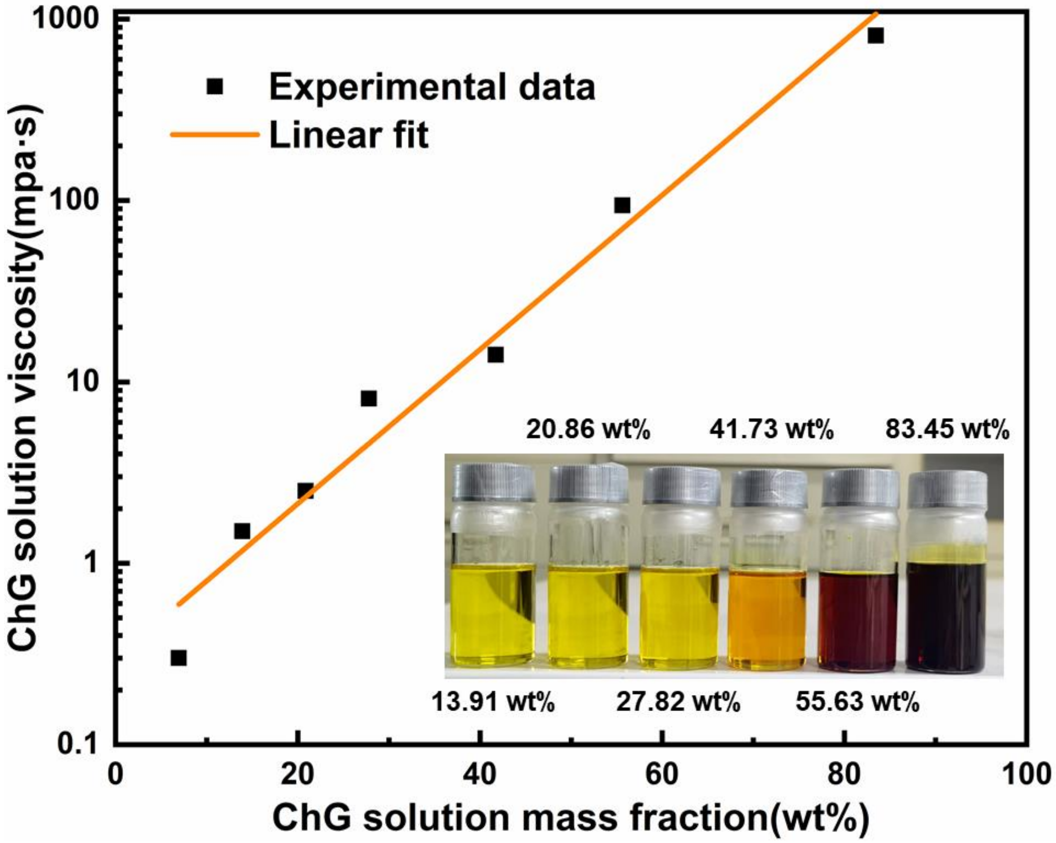

Figure S10. Viscosity of ChG solution with different concentrations. The illustration is an optical image of ChG solution with different concentrations. Related to Figure 4c.
